# Interleukin-2-330T/G and Interleukin-10-1082A/G Genetic Polymorphisms and B-Cell Non-Hodgkin Lymphoma

**DOI:** 10.4274/tjh.2018.0189

**Published:** 2018-11-13

**Authors:** Beuy Joob, Viroj Wiwanitkit

**Affiliations:** 1 Sanitation 1 Medical Academic Center, Bangkok, Thailand; 2 Honorary professor, Dr DY Patil University, Pune, India

**Keywords:** Interleukin, Lymphoma, Polymorphism

## To the Editor,

We read the publication “Association of Interleukin-2-330T/G and Interleukin-10-1082A/G Genetic Polymorphisms with B-Cell Non-Hodgkin Lymphoma (B-NHL) in a Cohort of Egyptians” with great interest [1]. Abdel Rahman et al. [1] concluded that “The present study highlights the possible involvement of the [interleukin (IL)] IL-2-330T/G genetic polymorphism in the susceptibility to [B-NHL] B-NHL in Egypt, especially indolent subtypes. Moreover, IL-10-1082A/G is not a molecular susceptibility marker for B-NHL in Egyptians” [1]. In fact, the role of polymorphism of IL is widely mentioned in relationship to NHL susceptibility [2]. We agree with the observation of Abdel Rahman et al. [1]. The differences of the effects of IL-2-330T/G and IL-10-1082A/G can be explained by molecular quantum calculations of molecular weight changes. This is the same phenomenon as seen in other polymorphisms and it can affect the clinical appearance of many medical disorders, such as the effect of CTLA-4 A49G polymorphism on autoimmune blood disease [3]. For IL-2-330T/G and IL-10-1082A/G, the change of molecular weight is equal to -107.07 and +16 per molecule, respectively. This means that a molecule with IL-2-330T/G requires more molecular mass and a molecule with IL-10-1082A/G requires less molecular mass to complete a biological process compared to a naïve molecule.
